# Prostate MRI and PSMA-PET in the Primary Diagnosis of Prostate Cancer

**DOI:** 10.3390/diagnostics13162697

**Published:** 2023-08-17

**Authors:** Lorenzo Cereser, Laura Evangelista, Gianluca Giannarini, Rossano Girometti

**Affiliations:** 1 Institute of Radiology, Department of Medicine, University of Udine, 20072 Milan, Italy; lcereser@sirm.org; 2 University Hospital S. Maria della Misericordia, Azienda Sanitaria-Universitaria Friuli Centrale (ASUFC), p.le S. Maria della Misericordia, 15, 33100 Udine, Italy; 3 Department of Biomedical Sciences, Humanitas University, Via Rita Levi Montalcini 4, Pieve Emanuele, 20072 Milan, Italy; 4 IRCCS Humanitas Research Hospital, Via Manzoni 56, Rozzano, 20089 Milan, Italy; 5 Urology Unit, University Hospital S. Maria della Misericordia, Azienda Sanitaria-Universitaria Friuli Centrale (ASUFC), p.le S. Maria della Misericordia, 15, 33100 Udine, Italy

**Keywords:** prostatic neoplasms, magnetic resonance imaging, radiopharmaceuticals, positron-emission tomography computed tomography, biopsy, neoplasm staging

## Abstract

Over the last years, prostate magnetic resonance imaging (MRI) has gained a key role in the primary diagnosis of clinically significant prostate cancer (csPCa). While a negative MRI can avoid unnecessary prostate biopsies and the overdiagnosis of indolent cancers, a positive examination triggers biopsy samples targeted to suspicious imaging findings, thus increasing the diagnosis of csPCa with a sensitivity and negative predictive value of around 90%. The limitations of MRI, including suboptimal positive predictive values, are fueling debate on how to stratify biopsy decisions and management based on patient risk and how to correctly estimate it with clinical and/or imaging findings. In this setting, “next-generation imaging” imaging based on radiolabeled Prostate-Specific Membrane Antigen (PSMA)-Positron Emission Tomography (PET) is expanding its indications both in the setting of primary staging (intermediate-to-high risk patients) and primary diagnosis (e.g., increasing the sensitivity of MRI or acting as a problem-solving tool for indeterminate MRI cases). This review summarizes the current main evidence on the role of prostate MRI and PSMA-PET as tools for the primary diagnosis of csPCa, and the different possible interaction pathways in this setting.

## 1. Introduction

Prostate cancer (PCa) is the second most common cancer type in men, with an incidence of 1.4 million new diagnoses per year and a global mortality of 350,000 deaths [1,2]. While most patients show organ-confined disease at the time of diagnosis and a life expectancy of up to 99% over ten years [3,4], the five-year survival rate drops to 30–40% in cases presenting local pathological lymph nodes or distant metastases [5]. To date, clinical tools for PCa diagnosis encompass digital rectal examination (DRE), serum prostate-specific antigen (PSA) testing, imaging techniques including transrectal ultrasound (TRUS) and magnetic resonance imaging (MRI), and biopsies [6]. In order to overcome the invasiveness of biopsy procedures, there is intense research on complementary diagnostic tools, e.g., the analysis of circulating serum and urine biomarkers [7] and the refinement of imaging-assisted strategies.

Among the available diagnostic tools, prostate MRI has emerged as the most effective imaging technique for the primary diagnosis of PCa [8]. When compared with systematic biopsy alone, MRI assists in adding targeted biopsies of suspicious imaging findings, thus, in turn, enhancing the detection of clinically significant PCa (csPCa), i.e., an International Society of Urogenital Pathology (ISUP) grading group of ≥2 cancer. However, MRI is not devoid of limitations, mainly represented by the disappointing false-positive rate [9], intermediate inter-reader agreement in interpretation [10], and dependence on readers’ experience [11]. Additionally, while diagnostic MRI serves for locoregional staging, this technique cannot perform distant staging as a guide for planning primary treatment.

In parallel with MRI, “next-generation imaging” has evolved rapidly. In particular, Positron Emission Tomography/Computed Tomography (PET/CT) with Prostate-Specific Membrane Antigen (PSMA) radiolabeled with 18F or 68Ga has significantly affected the management of patients with PCa, mainly in cases of biochemical recurrence of disease, with a substantial impact on the therapeutic management [12]. In recent years, many efforts have been made to understand the role of PSMA-PET also in the initial staging of disease and, more recently, as an imaging tool for the initial diagnosis of PCa. Indeed, PSMA-PET/CT has been proposed as a complementary tool in patients at intermediate-to-high risk of csPCa with negative or inconclusive MRI findings [13,14] or even as a replacement to MRI to identify the sites of target biopsy [15].

However, the role of PSMA-PET in primary diagnosis is still to be fully established, suggesting that interaction and integration with MRI are needed to optimize an imaging-guided diagnostic pathway maximizing the detection of csPCa while minimizing overdiagnosis of clinically insignificant PCa (ciPCa). In this review, we present an imaging-centered summary of the current role of both MRI and PSMA-PET in the primary diagnosis of csPCa. This review also presents possible interaction pathways, considering PET/MRI as a potential tool to provide the most effective interaction between MRI and PSMA-PET. For the sake of space, we will not discuss the role of ultrasound in guiding MRI-informed prostate biopsy or emerging imaging techniques that could contribute further to the diagnostic pathway of csPCa, e.g., micro-ultrasound. Those topics have been treated comprehensively elsewhere [16,17].

## 2. The Role of MRI

Reviewing the reported sensitivity, specificity, positive predictive value (PPV), and negative predictive value (NPV) for csPCa can help interpret the strengths and weaknesses of MRI and, in turn, its current clinical role. For the sake of space, we will not focus on the MRI technique, i.e., multiparametric MRI (MRI) vs. bi-parametric MRI avoiding contrast injection. We instead will refer to “MRI”, assuming that relevant results in this field have been achieved with both technical approaches, though multiparametric MRI is still recommended by the Prostate Imaging and Reporting Data System (PI-RADS) in most clinical scenarios [18].

### 2.1. Diagnostic Accuracy

NPV expresses the clinical impact of MRI at its best. According to the literature [19], the NPV of MRI approximates 91% for ISUP grade ≥ 2 PCa when considering PI-RADS 1–2 categories as “the negative examination”. NPV increases up to 97% for ISUP grade ≥ 3 PCa. A meta-analysis of studies applying PI-RADS v.2.1 categorization reported a pooled sensitivity of 90% for both the whole gland and the transitional zone [20]. It is worth emphasizing that the NPV is influenced by the condition’s prevalence [21]. Therefore, estimating the individual risk of csPCa is crucial to rely on a negative MRI result confidently, provided that the interpretation has been made by an adequately experienced reader [11]. The expected prevalence of csPCa (ISUP grade ≥ 2) in biopsy-naïve men, as derived from European studies, ranges from 28% to 49%, with an average of 39% [22]. In this typical scenario, MRI is expected to avoid unnecessary prostate biopsies in about one-third of biopsy-naïve men undergoing the examination [23], thus reducing biopsy-related costs and morbidity and, more importantly, decreasing the overdiagnosis and the risk of overtreatment of ciPCa [23,24,25,26].

**Figure 1:**
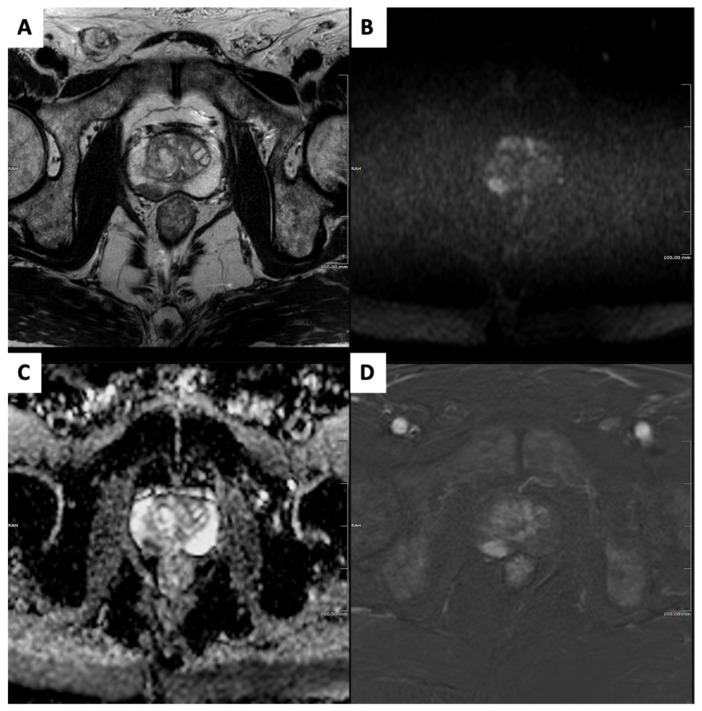
Clinically significant prostate cancer in a 75-year-old biopsy-naïve subject who underwent magnetic resonance imaging (MRI) for raised prostatic-specific antigen level (7 ng/mL) and suspicious digital rectal examination. Prostate MRI showed a 13 mm PI-RADSv2.1 category 4 focus in the right mid-gland peripheral zone, demonstrating homogeneous moderate hypointensity on axial T2-weighted image (**A**); focal-restricted diffusion with marked hyperintensity on the high b-value image (**B**) and marked hypointensity on the apparent diffusion coefficient map (**C**); and focal, early enhancement on a digitally subtracted, fat-saturated T1-weighted image from the dynamic contrast-enhanced sequence (**D**). Targeted cores from transperineal biopsy showed a ISUP grade 2 prostate cancer.

MRI shows a sensitivity greater than 90% for csPCa [23] (Figure 1). However, PPV and the specificity of MRI vary greatly in different studies, with pooled values ranging from 40 to 72% and 37 to 74% according to metanalysis, respectively [9,20,23,27]. These results suggest that the biopsies targeted to MRI findings harbor too many false-positive results, thus prompting the need for better stratification of the thresholds for triggering prostate sampling. PPV and specificity can vary based on the interpretation of PI-RADS 3 findings, which currently represent the threshold for auctioning prostate biopsy [28]. The pooled prevalence of csPCa is up to 25% only in the PI-RADS 3 category [29,30], suggesting that the rate of PI-RADS 3 calls can impact the accuracy of csPCa detection. In a multicenter cross-sectional study from Westphalen et al. involving subspecialized abdominal radiologists and 3449 subjects (38% of which were biopsy-naïve), the PPV of MRI was 35% for a PI-RADS v.2 score ≥ 3 and 49% for a PI-RADS v.2 score ≥ 4 [31]. Mazzone et al. found a 13% pooled PPV when categorizing index lesions as PI-RADS 3 [9]. Wadera et al. showed that including PI-RADS 3 findings among the “positive” results triggering biopsy determined a statistically significant reduction in specificity (33% vs. 71%, *p* < 0.001) [29]. The PI-RADS 3 call rate should be minimized, representing a benchmark for quality assessment and readers’ experience. The PI-RADS 3 call rate’s desirable value is less than 10% in high-volume centers [22].

In light of the disappointing false-positive rate of targeted biopsies [32], integrating MRI findings with clinical information is emerging as the main strategy to stratify patients’ risk of having csPCa and, in turn, biopsy decisions [33,34]. The most popular and easy-to-do approach involves the adjustment of the MRI findings with the prostate-specific antigen density (PSA-D) [35], an index that should be included in the MRI report according to the PI-RADSv2.1 [18]. PSA-D represents the ratio of serum PSA to prostate volume, with values exceeding 0.10–0.15 ng/mL/cc as predictive indicators for csPCa [36]. This index was proven effective as a tie-breaker, saving unnecessary prostate biopsies in men with PI-RADS 3 lesions and low PSA-D as opposed to increased cancer detection in men with PI-RADS 3 lesions and high PSA-D [27]. In a cohort of 123 biopsy-naïve patients, Girometti R. et al. [37] found that the benefit of stratifying MRI findings with PSA-D also extends to PI-RADSv2.1 category 4, which is the second major source of false-positives (up to 52% on a per-lesion basis in the literature [38]). The specificity for csPCa detection can improve from 54% to 72% and 86% when adjusting PI-RADS categories for PSA-D thresholds of 0.10 ng/mL mL^−1^ and 0.15 ng/mL mL^−1^, respectively [37]. Given the potential clinical impact of the PSA-D parameter, it is advisable to employ methodologies that ensure a precise and standardized prostate volume measurement, thus making its adoption desirable in clinical practice [28]. In this regard, artificial-intelligence-based tools for whole-gland segmentation may assist the radiologist in rapidly, reliably, and accurately evaluating prostate volume, thus replacing the traditional TRUS- or MRI-based techniques [39].

### 2.2. EAU Guidelines

**Figure 2:**
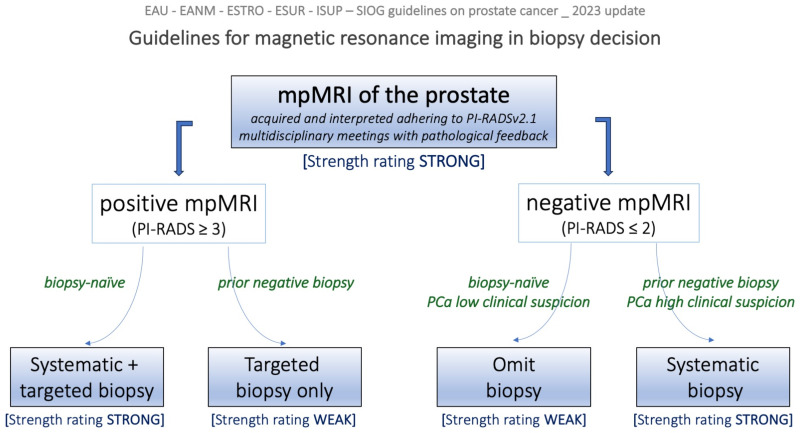
Decision tree diagram illustrating the role of magnetic resonance imaging in biopsy decision according to EAU-EANM-ESTRO-ESUR-ISUP-SIOG guidelines on prostate cancer, 2023 update. Please refer to the guidelines [28] for a more refined insight into strategies for risk stratification and biopsy decisions.

Guidelines from the European Association of Urology (EAU) recommend performing prostate MRI before biopsy in men with clinical suspicion of csPCa [28]. For this task, adherence to PI-RADS guidelines when acquiring and interpreting MRI is strongly encouraged, with PI-RADS category ≥ 3 findings triggering biopsy. Two biopsy strategies are promoted, the “combined pathway”, which consists of systematic plus targeted biopsies in biopsy-naïve patients, and the “MRI pathway”, when performing MRI-targeted biopsies in patients with a prior negative biopsy. After a negative MRI, i.e., an examination displaying PI-RADS category < 3 findings, systematic biopsy should be reserved for high-risk subjects only, especially if they have a prior negative biopsy. The two promoted biopsy strategies are illustrated in Figure 2.

The “combined pathway” puts into practice the “rule-in” ability, which is the capacity to maximize the detection of csPCa. Evidence supporting such a pathway derives from studies such as the MRI-FIRST [40] and PAIREDCAP [41], indicating that in biopsy-naive patients who undergo MRI, the most effective approach for diagnosing csPCa is a combination of targeted and systematic biopsy. Target cores can be acquired by fusing MRI images with real-time ultrasound guidance via cognitive or software assistance. This approach has been shown to increase the detection rate of csPCa [42,43,44,45,46,47,48,49] and improve risk stratification strategies based on volume and ISUP scores [43,44,45].

The “MRI pathway” focuses on the “rule-out” ability, which is the capacity to minimize the detection of ciPCa at the cost of missing a small proportion of csPCa. The use of such a pathway is substantiated by a sub-analysis of the FUTURE trial, which compared three different techniques of targeted MRI-informed biopsy in men with previous negative prostate sampling [50,51]. The analysis demonstrated that by excluding systematic biopsies in a repeat-biopsy scenario, only 1.3% of ISUP grade ≥ 2 cancers would have been missed [51]. The optimization of the “MRI pathway” must include quality control and assurance programs concerning all the chain links of the pathway, ensuring standardization and quality checkpoints of MRI technique and interpretation, as well as biopsy planning, acquisition, and interpretation [17,28,52].

The EAU guidelines suggest using individual-level risk profiling to balance the pathways’ inherent “rule-in” and “rule-out” capabilities. Factors such as aversion to biopsy or cancer diagnosis are usually considered [28]. Further studies are needed to assess the consistency of risk-adaptation strategies across different scenarios, encompassing varying prevalence of csPCa, as well as expertise levels of physicians and radiologists involved in the multidisciplinary team [33].

### 2.3. Open Questions

The ever-increasing use of pre-biopsy MRI raises two main issues. The first one is related to how cost-effective this use is. A recent systematic review [53] and a previous report compiled for the Canadian Agency for Drugs and Technologies in Health [54] showed that incorporating pre-biopsy MRI leads to better cost-effectiveness outcomes than ultrasound-guided biopsy only. This aligns with the expected cost reduction, e.g., from avoiding unnecessary biopsies in men with a negative MRI or less expansive treatments related to earlier diagnoses [53]. However, it is still difficult to determine which designs and methods of integrating pre-biopsy MRI into the diagnostic pathway offer superior cost-effectiveness, e.g., whether systematic biopsy can be omitted in biopsy-naïve men [53].

Second, it has been suggested that the current practice of adding target biopsies on MRI findings in biopsy-naïve patients is at risk of having no benefits on disease-specific mortality, but rather an unfavorable benefit-to-harm ratio compared with systematic biopsy alone [55,56]. According to statistical modeling on previous studies, Vickers [55] found a potential increase in men diagnosed and treated to prevent one single death from csPCa, i.e., potential MRI-induced overdiagnosis and overtreatment of cancers associated with low mortality if undetected by systematic biopsy [55,56]. This has been related to the risk of histological grade shift inherent in the use of MRI, i.e., the fact that targeting the biopsies to MRI findings makes the detection of the cancer zone with the highest ISUP grading group more likely compared with systematic biopsy; for example, a small volume Gleason score 3 + 4 lesion with a small “4” component has a greater probability of being classified as ISUP 2 (Gleason score 3 + 4) and treated accordingly compared with systematic biopsy (greater probability of classifying the lesion as ISUP 1 [Gleason score 3 + 3] to be referred to active surveillance) [55]. The strategy proposed by the ISUP 2019 consensus to mitigate grade shift is using an aggregated ISUP grading group summarizing the results of all biopsy cores from target biopsy rather than using the core with the highest ISUP grade [57]. However, the grade shift phenomenon further emphasizes the need to improve the knowledge of how to properly use MRI results in combined models of patient risk stratification and treatment decisions.

## 3. Role of PSMA-PET

The emerging role of PSMA-PET in the primary diagnosis of prostate cancer (PCa) can be derived from some recent reference studies, as discussed below.

### 3.1. Primary Staging

Many retrospective studies demonstrated that PSMA-PET has imaging advantages for defining the extension of disease compared with conventional imaging [58,59,60]. Since 2020, prospective randomized trials have established its role in this setting with a higher level of evidence [12,60,61,62,63]. The first study was published in 2020 by Hofman et al. [64]. The authors performed a randomized clinical trial, the proPSMA trial, to compare the diagnostic performances of PSMA-PET/CT vs. conventional imaging (both contrast-enhanced CT and bone scintigraphy) in patients with a high and very high risk of PCa. By including PSMA-PET in the diagnostic pathway, the authors found a significant increase in the area under the curve (AUC) (from 65% to 92%), a significant change in therapeutic management (from 15% to 28%), a reduction in equivocal findings (from 21% to 7%), and a reduction in radiation dose (from 19.2 to 8.4 mSv). Hope et al. [63] conducted a prospective multicenter single-arm open-label phase 3 imaging trial to evaluate the diagnostic efficacy of [68Ga]Ga-PSMA-PET/CT in 764 patients with intermediate-to-high-risk PCa. A total of 277 of the 764 (36%) subsequently underwent prostatectomy with lymph node dissection (efficacy analysis cohort). Based on pathology reports, 75 of 277 patients (27%) had pelvic nodal metastasis. The results of [68Ga]Ga-PSMA-PET/CT were positive in 40 of 277 (14%), 2 of 277 (1%), and 7 of 277 (3%) patients for pelvic nodal, extra pelvic nodal, and bone metastatic disease. Sensitivity, specificity, PPV, and NPV for pelvic nodal metastases were 40%, 95%, 75%, and 81%, respectively [63]. A cohort study (the OSPREY trial) was validated using [18F]F-PSMA-PET/CT in men with high-risk prostate cancer undergoing radical prostatectomy with pelvic lymphadenectomy. In 252 evaluable patients, 18F-PSMA-PET/CT had a median specificity of 97.9% and a median sensitivity of 40.3% among three readers for pelvic nodal involvement; median PPV and NPV were 86.7% and 83.2%, respectively [61].

Based on the available literature evidence, [68Ga]Ga or [18F]F-PSMA-PET/CT demonstrated a moderate sensitivity for the identification of lymph node metastases in high-risk patients, but a high specificity, thus enhancing the utility of the new generation of imaging as compared with the conventional one, although with limited results. Additional studies are ongoing for evaluating the impact of these imaging modalities from different points of view: cost-effective or cost-saving and quality of life or well-being.

### 3.2. Primary Diagnosis

**Figure 3:**
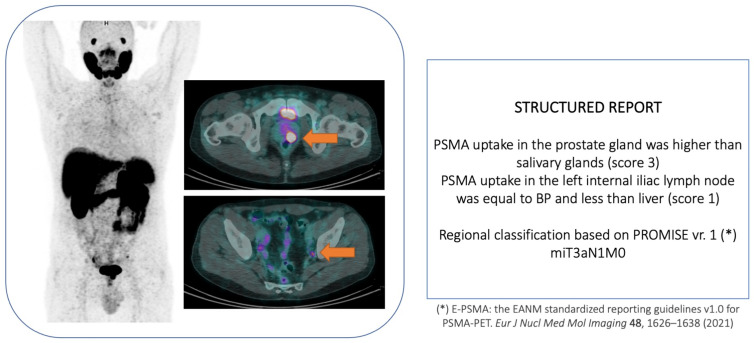
68Ga-PSMA-11-PET/CT scan in an ISUP grade 4 prostate cancer patient. PET/CT image demonstrated the presence of a large primary tumor in the left lobe of the prostate gland and moderate tracer uptake in a subcentimetric left internal iliac lymph node. The imaging staging was expressed in accordance with the PRIMARY score [69].

In recent years, increased attention has been given to using PSMA-PET as a diagnostic agent for detecting suspected prostate lesions. In the study by Liu et al. [65], the authors enrolled 31 patients with a previous negative prostate biopsy but persistent elevated serum PSA who underwent [68Ga]Ga-PSMA-PET/CT imaging before undergoing repeat prostate biopsy. A structured analysis was used to interpret the imaging: miPSMA score [66]. In the case of a negative 68Ga-PSMA-PET/CT (defined by the score: mi 0/1), patients were studied by a 12-core standard biopsy plus a 2-core target biopsy around the highest-uptake foci. Conversely, a positive PSMA-PET scan (defined as a score of mi 2/3) prompted a 12-core standard biopsy plus up to a 4-core target biopsy around positive focus/foci. Thirteen patients had a negative scan, and 18 had a positive one (41 vs. 59%, respectively). The sensitivity, specificity, PPV, and NPV for all PCa and csPCa lesions were 93% vs. 100%, 75% vs. 68.4%, 77.8% vs. 66.7%, and 92.3% vs. 100%, respectively. Later, Zhang et al. [67] compared the diagnostic accuracy of [68Ga]Ga-PSMA-PET/CT with trans-rectal ultrasound (TRUS) in two subsets of patients with suspected PCa (PSA level > 4 ng/mL). PSMA-PET was defined as positive or negative per the SUVmax of the index lesion [68]. PCa and csPCa detection rates were 43% vs. 31.6% and 40% vs. 25%, respectively, for PSMA-PET and TRUS. However, PSMA-PET detected significantly more cases of csPCa amongst patients with PSA 4.0–20.0 ng/mL than TRUS (27.02% vs. 8.82%), with the highest value of detectability in cases of PSA levels > 20 ng/mL (60.9% vs. 46.2%, respectively, for PSMA-PET and TRUS). Recently, Emmett et al. [69,70] conducted the PRIMARY trial, a prospective multicenter phase II imaging trial, which enrolled men with suspected PCa, no prior biopsy, and a recent MRI examination (6 months), for whom prostate biopsy was planned. Two hundred ninety-one patients were enrolled and sent to [68Ga] Ga-PSMA-PET/CT. The interpretation of the images was made per the PRIMARY score, which is a five-point scale from 1 to 5 (1 = no PSMA uptake or low-grade activity; 2 = diffusion transition zone activity, 3= focal transition zone activity; 4 = focal peripherical zone activity and 5 = PSMA SUVmax > 12). Based on the PRIMARY score, the detection rates for csPCa were 8.5%, 27%, 38%, 76%, and 100%, respectively, for points 1, 2, 3, 4, and 5. An example case is illustrated in Figure 3.

Recently, the PROMISE score version 2 was released. It integrates an updated miTNM system (PRIMARY score), improved assessment of local disease, and a slightly modified PSMA-expression score for clinical routine. Additional data about the response monitoring framework were defined by qualitative and quantitative parameters to be recorded for a longitudinal assessment in clinical trials [71].

Based on the three described papers, PSMA-PET seems promising in defining the PCa lesions. However, some criticisms emerged: (1) no standardized criteria for the evaluation of imaging interpretation, (2) the limited number of enrolled patients, and (3) the absence of phase III randomized clinical study. Nevertheless, some ongoing clinical trials on this topic aim to address clear information (Table 1).

The main advantages of the employment of PSMA-PET/CT or PET/MRI in the identification of PCa lesions in the early phase of disease would be: (1) more targetable diagnosis; (2) the opportunity to plan focal therapies, mainly in fragile patients or those with multiple comorbidities; and (3) to reduce unnecessary or serial biopsies that can be linked to potential side effects or complications. In this setting, PSMA-PET has the potential to answer an unmet clinical need.

## 4. Pathways of Interaction between MRI and PSMA-PET

**Figure 4:**
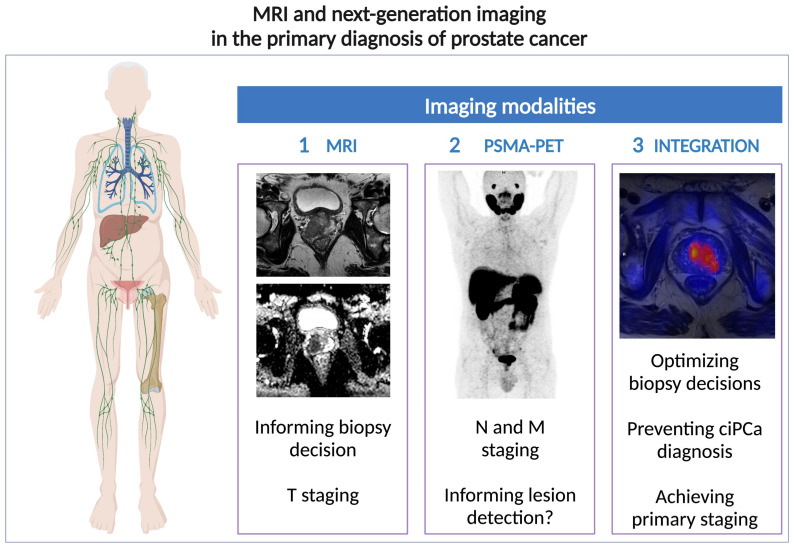
Current roles of prostate MRI, PSMA-PET, and integration imaging for PCa primary diagnosis.

Guidelines currently recommend using MRI for the primary diagnosis of PCa, reserving PET for staging, especially in high-risk localized disease/locally advanced disease [28], and detecting biochemically relapsed disease after radical prostatectomy [72]. Despite being deemed *“usually not appropriate”* for PCa detection based on the American College of Radiology (ACR) Appropriateness Criteria [73], the utilization of PET/MRI and PET/CT imaging has been explored as a potential substitute or adjunct to MRI for this purpose [74,75], as illustrated in Figure 4.

This section discusses the up-to-date literature on the integration of or “competition” between MRI and PSMA-PET for PCa detection, focusing on five possible pathways and including PET/MRI as a promising adjunctive tool in the “next-generation imaging” setting.

### 4.1. Pathway 1: “MRI vs. PET/CT”

The diagnostic accuracy of PSMA-PET/CT for ISUP grade ≥ 2 csPCa detection is close to that of MRI, with a pooled sensitivity, specificity, and accuracy of 97%, 66%, and 86%, respectively [76]. According to a metanalysis, the pooled negative likelihood ratio (NLR) was 0.05 [76], which is much lower than the NLR reported for MRI, which ranges from 0.16 to 0.26 [77,78,79,80]. NLR is a prevalence-independent test performance metric calculated by dividing the probability of a negative test result in subjects with a particular condition (i.e., csPCa) by the probability of a negative test result in subjects without the same condition [81]. Notably, a diagnostic test exhibiting an NLR below 0.10 is typically regarded as effectively ruling out the possibility that a subject has the disease [82]. This seems to hold true for PSMA-PET/CT, but not for MRI in the case of csPCa. Nevertheless, there is a lack of studies confirming this NLR value, as well as performing a head-to-head comparison between the NLRs of PSMA-PET/CT and MRI. Consequently, it remains challenging to draw definitive conclusions at this time.

Studies directly comparing PSMA-PET/CT and MRI in the diagnosis of csPCa showed conflicting results. Donato et al. [15] demonstrated an additional detection yield favoring PSMA-PET/CT over MRI for both index (14% vs. 4%) and total (18% vs. 5%) lesions, particularly showing incremental values in detecting secondary cancer foci and smaller cancers. This translated into a higher sensitivity (95% for PET/CT vs. 86% for MRI) without compromising specificity (94% for PET/CT vs. 95% for MRI). However, it should be noted that these findings may be overestimated due to the patient selection criteria. Since all subjects were triaged with MRI with subsequent MRI-oriented biopsy before PET/CT staging, most patients were deemed high-risk, with a high (47%) proportion of ISUP grade ≥ 3 csPCa. Other studies [83,84] reported similar detection rates for PSMA-PET/CT and MRI, approximating 90% on a per-lesion basis. Notably, in the study of Kalapara et al. [84], there was no additional value of PET/CT for the localization of transitional zone lesions (85% for PET/CT vs. 80% for MRI, *p* > 0.9). These lesions are considered challenging for MRI, with a reported sensitivity of 80% and a specificity of 85% [85].

### 4.2. Pathway 2: “PET/CT Following MRI”

In a study by Sonni et al. [83], the combination of PSMA-PET/CT and MRI improved tumor extent delineation and the identification of multifocal lesions, while not providing significant improvements over MRI alone for the T staging. Exterkate et al. [86] recently presented similar findings regarding T staging, wherein they observed no additional value of PSMA-PET/CT over MRI in evaluating extra-prostatic extension and seminal vesicle invasion.

The PRIMARY clinical trial [70] aimed to investigate the additive diagnostic value of combining MRI and localized (i.e., limited to the pelvis) PSMA-PET/CT for detecting csPCa in naïve-biopsy individuals. Results showed that the addition of PSMA-PET/CT to MRI improved the MRI sensitivity (83% vs. 97%) and NPV (from 72% to 91%), thus providing a more effective strategy for safely avoiding biopsy compared with relying on MRI alone [69]. Nevertheless, MRI sensitivity and NPV in this study were notably lower than benchmark values (pooled sensitivity of 90% and NPV of 91%) [19,23].

Hagens and van Leeuwen proposed a diagnostic imaging strategy [14] integrating MRI and PSMA-PET/CT to guide biopsy in biopsy-naïve individuals. According to their approach, MRI is reserved for individuals with intermediate-to-high risk based on clinical nomograms. When the MRI results are negative or equivocal, i.e., when it harbors PI-RADS category 1–3 findings only, the authors recommend referring to PSMA-PET/CT to target the biopsy sites. By applying the proposed strategy, individuals initially deemed low-risk or with negative PSMA-PET/CT following a negative/equivocal MRI can safely avoid undergoing a biopsy. This approach aims to reduce unnecessary invasive procedures while ensuring appropriate evaluation for individuals with higher risk profiles or suspicious findings on imaging. Notably, higher-risk subjects would undergo concomitant nodal (N) and distant metastasis (M) staging using PSMA-PET/CT, thus allowing for the full potential of PSMA imaging to be realized [13].

### 4.3. Pathway 3: “MRI vs. PET/MRI”

We believe that the inquiry of “Which imaging modality is superior between MRI and PSMA-PET?” lacks significance and is flawed by concerns about the available evidence. Indeed, a previous meta-analysis [87] demonstrated a significantly higher accuracy for the PCa detection of PET/MRI than MRI. However, it is noteworthy that the reported pooled sensitivity (60%) and specificity (89%) values of MRI differ from the established referenced values of higher sensitivity (approximating 90%) and lower specificity (ranging from 37% to 74%) for ISUP grade group ≥ 2 csPCa from studies involving MRI-informed biopsy strategies [9,20,23,27]. We found analogous discrepancies in more recent studies comparing MRI and PET imaging, which reported an MRI with a 56–68% sensitivity and a 91–92% specificity for csPCa [88,89]. The reasons for such discrepancies may be related to the retrospective nature, small study populations, and high frequency of region-based rather than lesion-based estimation methods of the available studies [87]. A possible explanation for the higher accuracy for the csPCa detection of PET/MRI when compared with MRI is the risk of patient selection bias due to the inclusion of a high proportion of subjects at intermediate- to high-risk, which frequently overexpress PSMA, thus making accuracy results prone to overestimation [90].

### 4.4. Pathway 4: “PET/MRI Following MRI”

Previous studies demonstrated the utility of performing PSMA-PET/MRI after MRI in selected cases [91]. This approach capitalizes on the higher specificity of PSMA-PET/MRI in detecting primary PCa (pooled specificity of 81%) to counterbalance the inherently high false-positive rate of MRI, thus enabling patient reclassification and potentially avoiding unnecessary biopsies [91]. A study from Ferraro et al. [90] showed similar results when performing PSMA-PET/MRI on selected subjects with persistently elevated PSA levels and PI-RADS category ≥ 3 findings on MRI. By considering PET/MRI as an integration of a preliminary MRI, this combined approach achieved a patient-based 90% accuracy in detecting csPCa. Notably, the specificity was almost twice that of the MRI-based PROMIS trial [92] (41% vs. 81%) while maintaining a comparable sensitivity (93% vs. 96%).

### 4.5. Pathway 5: “Prostate MRI within PET/MRI”

Increasing evidence suggests that incorporating data from PSMA-PET into integrated MRI may yield superior diagnostic performance compared with each modality alone [93,94]. In a study from Al-Bayati et al. [95], in patients undergoing simultaneous pelvic PET/MRI acquisition, nearly one-third of lesions (15/41) were classified as PI-RADS category 3 at MRI, thus posing decision-making dilemmas. PSMA-PET/MRI upstaged 6 of these 15 lesions as highly likely for csPCa. All of them were confirmed malignant at pathology, translating into an increase in diagnostic accuracy from 68% with MRI alone to 95% with PET/MRI.

It is worth noting that a recent study from Bodar et al. [96] comparing MRI and [^18^F]DCFPyL-PET/MRI in intermediate-to-high risk patients before robot-assisted radical prostatectomy did not show a superior performance of PET/MRI compared with MRI in terms of detection, local staging, or targeting prostate biopsies. Further studies are warranted, especially using PSMA radiotracers, to prove the added value of combining MRI with PET/MRI. Whatever the strategy, i.e., PET/MRI following MRI or embedding MRI within PET/MRI, several methodological considerations remain to be addressed, including determining the optimal combination of PET and MRI parameters, establishing recommendations for PET/MRI reporting strategies [97], and identifying the most effective hybrid imaging rules for targeted biopsies. Moreover, as far as we know, no studies evaluated the cost-effectiveness of diagnostic strategies combining MRI with PSMA-PET/MRI, either in isolation or in comparison with strategies that combine MRI with clinical or laboratory variables, e.g., PSA-D. Finally, the limited worldwide availability of hybrid PET/MR scanners represents a great challenge for delineating future clinical trials addressing this end-point.

## 5. Conclusions

MRI is currently the imaging technique of choice in the diagnostic pathway of csPCa. Negative MRI can avoid unnecessary prostate biopsies and the overdiagnosis of ciPCa, while positive MRI identifies sites for target biopsies, which has proven to increase sensitivity. The suboptimal false-positive rate of target biopsies emphasizes the need for better clinical stratification of imaging findings or the incorporation of imaging results into clinical models driving personalized biopsy decisions.

In this evolving scenario, PSMA-PET plays an ever-increasing role as a staging tool in patients at higher risk, though the effects on the oncological outcomes of earlier detection of distant metastases are still unclear. It is reasonable to assume that this role, which is complementary to MRI, will increase over the next years, as the superiority compared with conventional imaging (CT and bone scan) is clear. PSMA-PET, both as PET/CT or PET/MRI, has also been proposed as a cancer detection tool, thus opening different scenarios for competition or integration. The benefits of using PSMA-PET/CT for the primary diagnosis of csPCa are uncertain in the light of current evidence, although this topic is a matter of ever-increasing research. Defining how different examinations interact at their best is reasonably more effective than competing with prostate MRI for the same tasks. Further studies are needed to support combined MRI and PET/CT-guided pathways for optimizing biopsy decisions, preventing the diagnosis of ciPCa, and achieving primary staging.

## Figures and Tables

**Table 1:** Ongoing clinical trials aiming to assess the role of PSMA-PET in the diagnosis of prostate cancer (ClinicalTrials.gov, accessed on 14 June 2023).

Number of Trial	Radiopharmaceutical Agent (Study Phase)	Primary End-Point	Ongoing
NCT04116086	68Ga-PSMA-11 (not available)	To evaluate the possible role of PSMA-PET-CT in the early detection of prostate cancer, reducing the rate of unnecessary prostate biopsies, and correct staging of the disease and corresponding management in cases of prostate cancer.	Unknown
NCT05820724	18F-DCFPyl (phase II)	To determine if PSMA-PET imaging plus MRI improves the detection of clinically significant prostate cancer as compared with MRI alone.	Not yet
NCT05815316	18F-PSMA-1007 (phase II)	To evaluate the role of fully hybrid PET/MRI with 18F-PSMA and MRI as a one-stop approach for the diagnosis of clinically significant prostate cancer.	Not yet
NCT05160597	68Ga-PSMA-11 (phase I)	To use 68Ga-PSMA-11 PET/CT in patients with negative prostate biopsies.	Yes
NCT05154162 (PRIMARY 2)	68Ga-PSMA-11 (phase III)	To prove that the addition of PSMA-PET/CT is non-inferior to MRI for the detection of csPCa in men with PI-RADS 2–3 disease, while providing the advantages of reducing unnecessary biopsies and limiting to targeted-only TPPB.	Yes

## Data Availability

Not applicable.
